# Provincial Hospital Arrangements

**Published:** 1914-08-29

**Authors:** 


### Provincial Hospital Arrangements.

### AYLESBURY.

(*From our Special Correspondent.*)

The Royal Buckinghamshire Hospital at Aylesbury have offered to place thirty beds at the disposal of the military authorities for urgent and serious cases requiring surgical treatment. These beds will be provided in addition to the ordinary number of beds at the hospital.

The local Voluntary Aid Detachment are very busy in making arrangements for the equipment of a temporary hospital for 200 beds if required by the War Office. Promises are being obtained for the supply of all the materials necessary for the hospital, and, in addition, articles of clothing are being made by a representative committee of ladies. The men's detachment are enrolling a large number of new members, and frequent drills are being held.

The Soldiers' and Sailors' Families Association, of the County Branch of which Lady Rothschild is the president, with Lady Fremantle as honorary secretary, are already distributing funds to the families and dependent relatives of soldiers and sailors who are now serving with the regular Army, including also Reservists and Territorials.

### BARNSLEY.

(*From our Special Correspondent.*)

Every arrangement as far as can be foreseen has now been made for war casualties that may be drafted to the town. Dr. Trevor Howell has a very efficient class of lady members of the St. John Ambulance Association. He has also arranged, in conjunction with Mrs. Montague Stanhope, to have any consignment of patients conveyed from train by motor ambulances, and the bearers will consist of male members of the St. John Ambulance Association, under the care of Mr. W. A. Bellamy, superintendent of the Barnsley branch. The Prince of Wales's Fund is also being heartily supported. The South Yorkshire coalowners have decided to subscribe £20,000 toward it, as well as contributing £2,000 for the relief of sufferers in Belgium.

### CHATHAM DISTRICT.

(*From our Special Correspondent.*)

Chatham, being an important naval and military centre, is providing for casualties on a large scale. The Naval Hospital, a comparatively new building with extensive grounds, accommodates 1,000 patients. The wards, operation theatres, kitchens and other departments are most excellent and every latest improvement has been introduced. It is built in blocks connected by a wide corridor. To augment its accommodation, however, the Admiralty have requisitioned the "Welcome" Home for Soldiers and Sailors, where all the cubicle partitioning has been removed, the floors have been carefully stopped and treated, and the whole building, equipped with an operation theatre, has been transformed into a hospital capable of taking close upon 200 beds. The Royal Sailors' Home has been dealt with in the same manner, and the Infirmary at Sheerness is to be used also for wounded sailors. At the Royal Naval Barracks provision has been made for another 500 patients. The military hospital at Fort Pitt, to which a new wing has been recently added, is of a much older type as compared with the naval hospital. Here there is accommodation for over 300 beds. At St. Bartholomew's Hospital, Rochester, the local general hospital, 50 beds have been offered to the Army, and should the necessity arise the number of beds will be increased. Three members of its honorary and resident staff are on active service with the R.A.M.C. (T.F.). A fleet of motor omnibuses of the very latest type has been requisitioned by the authorities for transporting cases from Sheerness to the Chatham hospitals. Each omnibus has been fitted to take four patients.

It has been decided that during the war the "Territorial Force Voluntary Aid Detachment," the "British Red Cross" and the "St. John Ambulance Society" in the County of Kent should amalgamate and work as the "Kent Voluntary Aid Organisation." Dr. Yolland, who is the chief staff officer, is acting as Honorary Treasurer and

Honorary Secretary. Dr. Cotton is the County Director.

### COVENTRY AND WARWICKSHIRE HOSPITAL

(*From our Special Correspondent.*)

The war is affecting the work of this hospital in many ways. Two members of the honorary staff have been called up for active service with their regiments, and three residents have been accepted by the Government for service; one has already left and is now serving in the R.A.M.C., and the other two men expect to be called up at any moment. Four sisters and five staff nurses have also volunteered. The local medical profession have kindly volunteered to help the hospital in any emergency arising owing to the depletion of its staff. Twenty beds are being offered to the authorities through the Red Cross Society, and the work of the hospital is being concentrated with a view to future emergencies.

The local detachments of the Red Cross Nurses have been granted permission to attend the wards of the hospital to the extent of six daily for instruction.

All contracts have ceased, and counter prices are being paid for most supplies with the exception of groceries. It has been decided to defer sending out an appeal to wipe off the debt on the Extension Fund.

Two members of the honorary medical staff, Dr. Duncan Davidson and Mr. F. Sydenham, have been called up, and the senior surgeon, Mr. Anderson, is with the R.A.M.C. Dr. Jones, junior house-surgeon, and Dr. Roberts, junior house-physician, are expected to be called up at any time, as their services were recently accepted.

### CROYDON.

(*From our Special Correspondent.*)

The committee of the Croydon General Hospital have offered to devote the "Mark" and the "Luke" wards, which consist of about forty beds, entirely for the treatment of sick and wounded soldiers and sailors. Admissions are being restricted to urgent and necessitous cases.

### DONCASTER AND DISTRICT.

(*From our Special Correspondent.*)

Lying within easy reach of the East Coast, and in ready communication by railway with the Humber ports, Doncaster is sure to be called upon to provide accommodation for the sick and wounded. The Doncaster Royal Infirmary badly needs extension, and so the authorities of the infirmary will not find it easy to place any appreciable number of beds at the disposal of the War Office. A similar state of affairs exists at the Poor-Law Institution at Balby, on the outskirts of Doncaster. The infirmary is congested, and the Doncaster Guardians have called in an architect to advise them on extension. The Guardians are not making any move until they are officially approached. The St. John Voluntary Aid Detachment officials have secured the Mansion House as a hospital for fifty beds ready for occupation; also in three large unoccupied houses a further fifty beds can be equipped. As convalescent homes the detachment has received offers of accommodation at Hickleton Hall (Lord Halifax's seat), Wyndthorpe (Lord Chetwynd), and the Hall, Bolton-on-Dearne, all within easy motoring distance of Doncaster. Although there is no local men's Voluntary Aid Detachment there are ample supplies of trained ambulance men from the adjacent collieries, upwards of forty of whom can be brought on the scene, with full equipment, whenever required. There is also a Voluntary Aid Detachment at Loversall, about three miles from Doncaster, where at about four hours' notice thirty beds can be made ready. The detachment is now staffing a "rest station" in one of the rooms of the engineers' department at Doncaster Station, and their services have already been in request on behalf of the troops under mobilisation in the neighbourhood.

### FELIXSTOWE.

(*From our Special Correspondent.*)

The Red Cross Hospital is in perfect order. A trained staff is ably assisted by members of the Voluntary Aid Detachment; everything is thoroughly organised for the reception of the sick and wounded. At present both the military hospital and the Red Cross hospital are caring for cases of ordinary camp casualties and sickness. The local cottage hospital has offered the use of its *x*-rays apparatus.

### GLOUCESTER.

(*From our Special Correspondent.*)

The two City Red Cross Detachments, under the command of Mrs. Lee-Williams and Mrs. Evans, have been ordered to hold themselves in readiness to open a hospital for fifty sick and wounded soldiers and sailors if necessary. As it was understood that a hospital of 100 beds would be preferable to two of fifty beds each, the Detachments have decided to join forces. After a considerable amount of trouble in obtaining a suitable building owing to the refusal of the Board of Education, who in the interest of the children had forbidden the use of the schools as hospitals, the Guardians have offered the use of the new infirmary to the Red Cross Detachments. The work of the building (which is not yet completed) is being hurried forward, and it is hoped that it will be ready for use early next week. The staff of the combined Detachments will consist of five trained nurses, thirty-two Red Cross workers, two surgeons, Dr. St. John and Dr. Washbourn, and two physicians, Dr. Terry and Dr. Alcock.

### GRAVESEND: AN EMERGENCY TEST.

(*From our Special Correspondent.*)

The preparations at Gravesend Hospital have been completed, so that all is ready to receive at any moment fifty or sixty wounded. Such are the arrangements that civil work will proceed as usual. In case it is necessary, the out-patients'

waiting hall will be converted into a ward, and a test as to the time it would take to do this was made on Friday week. The hall was cleared and beds and so forth duly installed, and all was ready within the space of half an hour. All the necessary equipment, which is "standing by," is from voluntary sources. Among the offers of assistance which Mr. Pearson, the hospital secretary, has received is a very valuable one from the President, the Earl of Darnley, who offers beds at Cobham Hall for convalescent patients. The same offer is made to St. Bartholomew's Hospital, at Rochester.

### LANCASTER.

*(From our Special Correspondent.)*

The Royal Lancaster Infirmary has offered thirty beds to the Government for the treatment of sick and wounded soldiers, and to make it possible the day rooms have had to be fitted up as hospital wards, and more beds than usual have been put temporarily into the ordinary wards.

The offer to place the Friends' Hall at the disposal of the committee for a temporary hospital has been gratefully accepted. This will enable the committee to draft convalescents to that hall from the infirmary proper, and so make room for urgent cases.

The military authorities have taken possession of the Bowerham Council buildings, Lancaster, and are fitting it up as a hospital. The teachers and scholars have had to be housed in the Sulyard Street School, which was closed a short time ago by order of the Board of Education.

Members of the Lancaster Medical Book Club have agreed to treat, free of charge, the wives and children of local Territorials who have volunteered, and to look after the patients of their colleagues called up for service.

### LEAMINGTON.

*(From our Special Correspondent.)*

The Warneford General Hospital have offered fifty beds to the War Office, and by re-equipping the empty wards it is assured that there will be no reduction in the number of beds usually available. The extra bedsteads have been promised by local tradesmen, and there has been a ready response for equipment. One of the members of this committee, Mrs. Emmet, of Moreton Paddox, has generously undertaken to furnish and maintain at her country residence fifty beds for convalescents, with a staff of medical men and trained nurses, the patients to be those of the sick and wounded who have been treated at the hospital in the first place, or from any other hospital taking men from the Army or Navy which applies through the Warneford Hospital. The local Ambulance Brigades are being specially trained for any emergency in stretcher work so as to bring the cases with as little pain as possible from the railway stations. Motor lorries and ambulances have also been requisitioned.

The committee have agreed to take probationers from the British Red Cross Society, eight for a month at a time. This has the approval of the honorary county director, Brig.-General Quayle Jones, C.B., who recommends the ladies to the hospital matron for appointment by her. Lectures are also being given weekly by the senior honorary physician, Dr. Cole, to about thirty Red Cross members.

### SALISBURY INFIRMARY.

*(From our Special Correspondent.)*

Fifty beds have been promised for the reception of soldiers, if required. To meet this need a ward long closed will be reopened, and extra beds will be added where space admits—the wards are fortunately very lofty and roomy—so that the needs of the civil population, which will certainly not be less in the near future, may also be met satisfactorily.

One ward has already been set aside for patients from amongst the large number of men stationed on the Plain, and some have been admitted.

### ST. LEONARDS, HASTINGS.

*(From our Special Correspondent.)*

The East Sussex Hospital has offered to the War Office forty beds, and the Buchanan Hospital, St. Leonards, from fifteen to twenty. There have been numerous private offers. As regards the Eversfield Chest Hospital, St. Leonards, the eldest son of Dr. Bullock, the assistant medical officer, who was one of the resident medical officers at St. Mary's, volunteered with other members of the surgical staff for active service, and is now on the Continent.

### WAKEFIELD AND DISTRICT.

*(From our Special Correspondent.)*

The War Office have now formally accepted the offer of two wards of twenty beds each at the Wakefield Clayton Hospital. The Voluntary Aid nurses have now practically completed the equipping of these beds, and it is hoped that they will be inspected and passed shortly. Eleven voluntary nurses daily attend at the hospital, two in each ward and three in the out-patient department. The West Riding County Council have placed their new Constabulary building, which is admirably adapted for the purpose, at the disposal of the St. John Ambulance Association as a hospital, and, as the Wakefield Board of Guardians have also offered sixty beds at the Wakefield Workhouse Infirmary, we may say that Wakefield has now provided as many beds as will be required. One of the honorary physicians at the hospital has been appointed with others to make arrangements for the conveyance of wounded from the coast hospitals to the inland hospitals.

The committee of the Wakefield Clayton Hospital have now considered arrangements for the feeding of the wounded or convalescent patients when they arrive.

### WOLVERHAMPTON.

*(From our Special Correspondent.)*

The board of management of Wolverhampton and Staffordshire General Hospital have offered to give some practical training to members of the County Branch of the Red Cross Society. This offer has been accepted, and for the last fortnight these ladies have been receiving practical training. The training is for twelve days of four hours each day. In addition the board have offered to the British Red Cross Society the use of thirty beds.

The Wolverhampton Board of Guardians, on the motion of Mr. S. R. Rhodes, chairman of the board, have also offered to the society as many beds in their Infirmary as may be available at the time, and perhaps this will mean 100 beds. Three members of the infirmary staff, including the night mental attendant, have joined their regiments. Eleven other officers may be called upon. Further, the Wolverhampton Parks and Baths Committee of the Corporation have offered the use of the baths with the idea of it being converted into a hospital for the use of the Society should it be required, and have promised to let them have it at forty-eight hours' notice.

Five members of the General Hospital honorary medical staff are already on military duty, eight members of the nursing staff, including the matron, Miss Hannath, have already left, and six others, being members of the Territorial Force, are holding themselves in readiness to join their units at short notice.

